# Development and testing of an informative guide about palliative care for family caregivers of people with advanced dementia

**DOI:** 10.1186/s12904-020-0533-3

**Published:** 2020-03-12

**Authors:** Lina Riedl, Manuela Bertok, Julia Hartmann, Julia Fischer, Carola Rossmeier, Andreas Dinkel, Marion Ortner, Janine Diehl-Schmid

**Affiliations:** 1 School of Medicine, Department of Psychiatry and Psychotherapy, Technical University of Munich, Ismaninger Str. 22, 81675 Munich, Germany; 2 School of Medicine, Department of Psychosomatic Medicine, Technical University of Munich, Langerstr. 3, 81675 Munich, Germany

**Keywords:** Palliative care, Advanced dementia, Involvement in care decisions, Decision making

## Abstract

**Background:**

Since people with advanced dementia are usually not able to make complex decisions, it is usually the family caregivers, as proxies, who have to decide on treatments and their termination. However, these decisions are difficult for the caregivers to make, as they are often inadequately informed and cannot properly assess the consequences; moreover, they are concerned about harming the sick person. We aimed to first develop an informative booklet about palliative care issues for caregivers of people with advanced dementia. Secondly, we aimed to investigate a change in family caregivers’ knowledge regarding palliative care issues and caregivers’ involvement in medical and care decisions before and after studying this booklet.

**Methods:**

A first version of the booklet was drafted by an experienced psychiatrist and palliative care specialist based on existing booklets and guidelines; necessary cultural adaptions were taken into consideration. A nominal group process was conducted to develop the informative guide. In order to investigate the acceptance of the booklet and the possibility to implement it, 38 patient-caregiver dyads were recruited, and caregivers were interviewed both before receiving the booklet and after 3 months of receiving the booklet.

**Results:**

Experts from various disciplines collaborated on a German booklet for family caregivers of people with advanced dementia as an information aid regarding issues of palliative care. The subsequent test showed that all caregivers had experienced a personal benefit from the booklet. Caregivers had a significant gain of knowledge after provision of the booklet. A large proportion of caregivers who had not previously considered and/or discussed medical topics reported that they had done so within 3 months after obtaining the booklet, or planned to do so in the near future.

**Conclusions:**

The caregivers valued the comprehensible, concise and well-structured information guide on palliative care issues in advanced dementia. They agreed it increases knowledge and prompts decision making and therefore should be developed in many languages and disseminated among family caregivers of people with dementia.

**Trial registration:**

clinicaltrial.gov, NCT03548142. Retrospectively registered 7 June 2018.

## Background

Dementia is a life limiting disease [1]. Worldwide, around 50 million people have dementia, and there are nearly 10 million new cases every year [2]. Advanced stages of dementia are characterized by severe cognitive and neurological impairments. Psychological and behavioral symptoms often occur [3]. Frequent dementia-associated medical complications include swallowing disorders, aspiration pneumonia, infections and dehydration [4]. The advanced and final, fatal stages of dementia are undoubtedly an indication for palliative care measures - as emphasized by the WHO [5].

In the advanced stages of dementia, as well as at end of life, it is often necessary to make significant medical treatment decisions such as tube feeding, hospital admissions, intensive care treatment, intravenous antibiotic administration, intravenous hydrogenation, or cardiopulmonary resuscitation. Since people with advanced dementia are no longer able to make complex decisions [6], the close relatives, as proxies, usually have to decide on treatments and interventions or - if indicated - their termination. These decisions are based on the person’s living will, if it is precise enough, or the person’s presumed will. However, family caregivers often find it difficult to make medical decisions because they do not feel that they have sufficient knowledge in order to adequately assess the consequences of their decisions; due to this lack of information, caregivers fear harming their loved ones [7, 8].

Currently, 1.7 million people with dementia live in Germany and approximately 300,000 new cases occur every year [9]. There is a scare amount of available information regarding end of life issues for caregivers of people with advanced dementia [10–12]. The WHO emphasizes the need for support for caregivers of people with dementia [13]. Booklets could help to provide the necessary support through increased knowledge and associated gain in competence. The aims of our study were to develop an informative booklet regarding palliative care issues for caregivers of people with advanced dementia as well as to subsequently investigate family caregivers’ knowledge of palliative care issues and their involvement in medical and care decisions before and after studying the booklet. We wanted to determine whether the booklet aids caregivers in learning about relevant issues and whether it provides the necessary self-confidence in order to take an active role in the decision making process.

## Methods

### Study phase 1: development of the booklet

A preliminary version of the booklet in the German language, entitled “*Advanced dementia and end of life - a guide for caregivers on the aims and options of palliative and hospice care”,* was developed by an experienced psychiatrist and palliative care specialist for relatives of people with advanced dementia. Existing booklets were considered in the development of the current informational guide [11, 14]. After the preliminary version of the booklet was drafted, a nominal group process was employed [15] with a panel of 19 experts from all relevant disciplines including gerontopsychiatry, neurology, palliative care, nursing care, ethics, and legal studies. The administrator of a long-term care facility, a representative of the German Alzheimer’s Society and the husband of a patient with severe dementia completed the expert panel. The experts were key persons within their respective disciplines who consented to participate in study. The participants were invited to attend the expert panel via email. All agreed to participate.

A first draft of the booklet was emailed to the experts with the request to review. All suggested changes were incorporated and the second draft was emailed to the experts. During a face-to-face meeting of the experts 6 weeks later, all controversial contents were discussed in detail until a consensus was found. Afterwards, the revised version of the booklet was emailed to the experts. All experts agreed on this final version and the consented version of the booklet (please see Table 1) was then provided to 20 persons who further simplified the language. These persons were laypeople who were recruited from acquaintances of the coauthors and nurses of a psychiatric hospital who were not involved in dementia care. It was ensured that people from all educational backgrounds were included. The laypeople were neither caregivers nor people with dementia and they did not necessarily have experience with dementia. They gave individual feedback in person or via email.

**Table 1:** Contents of the booklet

1) Information on the clinical symptoms in advanced stages of dementia and causes of death	
2) Goals of palliative care: when, where and how to seek palliative care support	
3) Legal basis of decision making including the role of the patient and their advance directives as well as the role of the legal representative and the doctor in ethical problems	
4) Life prolonging measures, i.e. tube feeding, admission to hospital, intensive care treatment, resuscitation; consequences for the patient	
5) Pharmacological and non-pharmacological treatment options for dyspnea, pain, agitation, anxiety and delirium; thirst and hunger at end of life	
6) Dying, death and after death; if applicable, advantages and disadvantages of different options are explained.	
A checklist assists the reader in ensuring that all necessary issues have been taken into consideration.	
An overview of the common drugs used in palliative care completes the booklet	

### Study phase 2: testing the booklet

In order to test the feasibility of the booklet, we chose a pre-post-design with a single arm trial (without control group). Results of a sample size estimation suggested the minimum recruitment of 30 patients with advanced dementia and their family caregivers in order to be able to detect a significant increase in caregivers’ perceived “involvement” in care decisions, as measured with a modified version of the *Patients’ Perceived Involvement in Care Scale* [16].

#### Impact of the booklet

To find out if the booklet improved knowledge and perceived involvement in care, two measures were applied at both visits:

##### Caregivers’ knowledge gain

To evaluate whether the booklet increased caregivers’ knowledge about palliative care issues. Caregivers self-rated their knowledge about 6 topics at both interviews (1 = good; 2 = moderate; 3 = poor), see supplementary material (S2).

##### Caregivers’ perceived involvement in care decisions

In order to evaluate the booklet’s impact on the caregivers’ involvement in care decisions, a questionnaire based on the *Patients’ Perceived Involvement in Care Scale* [16] was developed. In order to quantify the extent of caregivers’ participation in decision making, a score was derived from the caregivers’ answers (0 = no; 1 = yes) for questions 3a, 3b, 3c, 3d, 4, 5 and 6 (minimum score 0; maximum score of 7 indicates a “yes” answer for all questions). Questions are displayed in supplementary material (S3). Caregivers who had not considered or discussed topics, or made decisions before *Interview 1* and not at *Interview 2,* were asked if they plan to do so within the next 3 months.

### Participants and recruitment

In order to include at least 30 patient-caregiver dyads in the study while considering potential drop-outs, we aimed to recruit 38 dyads in order to answer our research questions. Patients were identified from the database of the outpatient Center for Cognitive Disorders of the Department of Psychiatry and Psychotherapy at the University Hospital of Technical University of Munich. The database, which consists of patient data for all patients that were examined at the center, was searched for subjects that were diagnosed with dementia between 2005 and 2010. Patient inclusion criteria were: advanced dementia with a legal representative holding power of attorney. From the 211 patients whose family caregivers could be reached by telephone, 53 did not fulfill the inclusion criteria, 81 had already died, four had a fatal somatic disease (unspecified in two cases, severe stroke in one case, hip fracture with complications in one case) and 35 did not wish to participate. A first visit to the patient’s home was arranged by telephone with those who were willing to participate.

### Procedures

After receiving written informed consent from the family caregiver who was also the patient’s legal representative, the dyads were included in the study.

#### Visit 1

At the first visit from the person with dementia, an experienced old-age neurologist and psychiatrist performed a standardized, detailed examination of the patient using standardized questionnaires including the Mini-Mental-Status Examination (MMSE) [17] and the Clinical Dementia Rating Scale (CDR) [18]. The duration of the examination depended on the severity of the dementia and lasted between 30 and 40 min.

Furthermore, an interview of the family caregiver was performed. The details are provided (please see supplementary material, S1). The duration of the interview lasted about 90 min. After *Visit 1*, the booklet was provided to the caregivers. A brief overview of the booklet content was given and the caregivers were asked to read the booklet before *Visit 2*, which was scheduled 3 months after *Visit 1*.

#### Visit 2

A second, detailed interview of the caregiver was performed at *Visit 2* 3 months later. For details regarding the second interview please also see supplementary material (S1).

##### Comprehensibility of the booklet

In addition to the interview at *Visit 2*, caregivers were asked to rate the booklet’s comprehensibility according to a standardized questionnaire [19] and the overall perceived benefit. The questionnaire is displayed in supplementary material (S4). The duration of *Visit 2* varied between 45 to 90 min.

### Ethics

The study was positively evaluated by the Ethics Committee of the Medical Faculty of Technical University of Munich (# 155/17 S). Written informed consent of the person with dementia and/or the legal representative and the family caregiver was obtained. The study is registered in ClinicalTrials.gov (NCT03548142).

### Statistics

Data were analyzed using Statistical Package for the Social Sciences (SPSS) 25. For quantitative data, means and standard deviation as well as minimum and maximum values were reported. The Wilcoxon signed rank test was used as nonparametric test for paired data. Because of the exploratory nature of this study, corrections for multiple testing were not performed. Significance level was set at 5%.

## Results

### Study phase 1: development of an informative guide, a booklet

During the development of the booklet, a few features considered specific for Germany were emphasized, including legal and cultural aspects:

The legal basis of treatment decisions for people who are unable to consent due to advanced cognitive impairment is an important part of the booklet. As a special condition of the German law, a person who is unable to consent needs to have a legal representative. A legal representative can either be a person who was authorized in advance by the (at that time consentable) patient or, if there is no such person, a court-appointed counselor. Many people in Germany are not aware of these regulations. The fact that a therapy decision cannot be made by close relatives as proxies underlines the importance of care planning in advance for these cases in which one becomes incapable of consent.

Due to Germany’s history (Holocaust involving the murder of mentally ill people, euphemistically deemed “euthanasia”), the social and political debate [20] about euthanasia is conducted more cautiously and reluctant - unlike, for example, in the Netherlands or Switzerland. Therefore, special attention was given to the wording in sections of the booklet concerning treatment options at the end of life.

Finally, since Germany is characterized by an increasing religious diversity, specific options of religious support were not included in the booklet. However, the need for spiritual support was stressed.

### Study phase 2

#### Patients’ characteristics

Out of the 38 patients with Alzheimer’s dementia (94%), frontotemporal dementia (3%) and dementia of multifactorial etiology (3%), 66% were female and 34% male. Mean age was 78.1 years (±10.3; min: 50. max: 100) at the time of *Visit 1*. 63% of the patients lived at home, 37% in a long-term care facility. The mean MMSE score was 5.2 out of 30 points (± 8.2; min: 0; max: 23). Severity of dementia was assessed as moderate (CDR = 2) in 32% and severe (CDR = 3) in 68% of the patients.

#### Family caregivers’ characteristics

At *Visit 1* the mean age of the family caregivers was 66.2 years (±15.0; min: 30, max: 87), 53% of the caregivers were female, 47% male. 58% of the relatives were spouses or life partners of the patients, 34% children and 8% other relatives or friends. On average, caregivers had 15.0 ± 3.61 years of education (school plus apprenticeship/ university). 50% of the caregivers lived together with the patient in the same household, 50% did not. 61% of the caregivers were retired.

Two of the 38 relatives were not visited a second time because they said they were unable to read the material due to time constraints.

### Impact of the booklet

#### Caregivers’ knowledge gain

Mean sum score of caregivers’ self-rated knowledge at *Visit 1* was 10.12 ± 2.78 (min: 6 reflecting good knowledge; max: 16 reflecting poor knowledge). At *Visit 2,* the mean sum score was lower with 7.69 ± 2.34 (min: 6; max: 16). A Wilcoxon signed rank test revealed a significant knowledge gain reflected by a decline in the sum score at *Visit 2* (z = − 4.427; *p* = 0.000; *n* = 36). In detail, caregivers experienced a gain of knowledge for all topics but most of all for the topic “Palliative/ hospice care services”. The knowledge difference was statistically significant (p between 0.000 and 0.033) for all single items except “Dying, death”. For detailed results see Table 2.

**Table 2:** Caregivers’ self-rated knowledge about various palliative care knowledge prior to and after provision of the booklet (1 = good; 2 = moderate; 3 = poor)

	Knowledge prior to provision of booklet	Knowledge after provision of booklet
1. Symptoms, course of dementia, prognosis	1.42 ± 0.60	1,17 ± 0.45
2. Goals of palliative care; palliative/ hospice care services	2.24 ± 0.82	1,44 ± 0.65
3. Tasks of legal representative	1.55 ± 0.67	1,17 ± 0.38
4. Life prolonging measures: e.g. tube feeding admission to hospital, resuscitation; consequences for the patient	1.66 ± 0.71	1,14 ± 0.49
5. Pharmacological, non-pharmacological symptom relief (e.g. dyspnea, pain, agitation, anxiety, delirium)	1.84 ± 0.71	1,42 ± 0.60
6. Dying, death (what is to be expected when the patient is dying)	1.57 ± 0.65	1,36 ± 0.59
**Sum Score**	10.12 ± 2.78	7.69 ± 2.34

#### Caregivers’ perceived involvement in care

The majority of the caregivers had already carefully considered different palliative care topics and taken an active role in the decision making process before *Visit 1* (Table 3, column A).

**Table 3:** Participatory decision making of the family caregivers

		A) Percentage of caregivers who answered “no” at *Visit 1*	B) Percentage of caregivers who answered “no” at *Visit 1* but participated in decision making before *Visit 2* (or planned to do so soon) in parentheses: “yes” or “yes, I plan to participate soon” at *Visit* 2/ “no” at *Visit 1*
	**1. I carefully thought about the following topics:**		
**Considerations**	1a. Symptoms, course of dementia, prognosis	**3% (1/36)**	100% (1/1)
	1b. Goals of palliative care; palliative/ hospice care services	**61% (22/36)**	72% (16/22)
	1c. Life prolonging measures: tube feeding admission to hospital, resuscitation; consequences for the patient	**11% (4/36)**	75% (3/4)
	1d. Pharmacological, non-pharmacological symptom relief (e.g. dyspnea, pain, agitation, anxiety, delirium)	**20% (7/36)**	14% (1/7)
	1e. Dying, death (what is to be expected when the patient is dying)	**11% (4/36)**	50% (2/4)
**Level of information exchange**	**2. I discussed following topics with the doctor/ care team:**		
	2a. Symptoms, course of dementia, prognosis	**17% (6/36)**	50% (3/6)
	2b. Goals of palliative care; palliative/ hospice care services	**81% (29/36)**	58% (17/29)
	1c. Life prolonging measures: tube feeding admission to hospital, resuscitation	**28% (10/36)**	10% (1/10)
	2d. Pharmacological, non-pharmacological symptom relief (e.g. dyspnea, pain, agitation, anxiety, delirium)	**26% (9/35)**	67% (6/9)
	2e. Dying, death (what is to be expected, when the patient is dying)	**42% (15/36)**	67% (10/15)
**Caregiver participation in decision making**	**3. I made a decision regarding the following topics:**		
	3a. Goals of palliative care; palliative/ hospice care services	**67% (24/36)**	67% (16/24)
	3b. Life prolonging measures: tube feeding admission to hospital, resuscitation; consequences for the patient	**28% (10/36)**	80% (8/10)
	3c. Pharmacological, non-pharmacological symptom relief (e.g. dyspnea, pain, agitation, anxiety, delirium)	**26% (9/35)**	67% (6/9)
	3 d. Dying, death (what is to be expected when the patient is dying)	**42% (15/36)**	60% (9/15)
	4. I suggested a certain kind of treatment/ care to the doctor/ care team	**42% (15/36)**	40% (6/15)
	5. I expressed doubts about treatment/care that the doctor/ care team suggested	**47% (17/36)**	18% (3/17)
	6. I gave my opinion (agreement or disagreement) about treatment and care	**17% (6/3)**	33% (2/6)

At *Visit 1*, the mean score derived from the caregivers’ answers regarding their participation in decision making was 4.5 ± 1.74 (min: 0; max: 7). The mean score at *Visit 2* after provision of the booklet was 3.4 ± 2.01 (min: 0; max: 7) and therefore significantly lower than at *Visit 1* (Wilcoxon signed rank test: z = − 2.955; *p* = 0.03; *N* = 36). This result reflects that many of the caregivers who had already made decisions before *Visit 1* did not make any further decisions after provision of the booklet. This ceiling effect at *Visit 1* prompted further analyses: We identified the percentage of caregivers who had answered the individual questions with “no” at *Visit 1;* that is, we identified how many caregivers neither considered nor discussed topics, nor made decisions (Table 3, column A). In order to find out whether the booklet influenced caregivers’ involvement in decision making, we investigated how many of these caregivers answered with “yes” at *Visit 2*, indicating that they had then considered or discussed topics, or made decisions (Table 3, column B).

Taken together, 14 caregivers considered, discussed and decided palliative care issues or planned to do so after provision of the booklet (100% of these caregivers had not done so before *Visit 1*).

#### Comprehensibility of the booklet

At *Visit 2*, 97% of the caregivers rated the booklet as comprehensible, concise and well-structured. The overall response to the booklet was very positive. The question, “How do you rate your personal benefit from the booklet?”, was answered with “high” by 69% and “moderate” by 31% of the caregivers.

## Discussion

Although the WHO underlines the need for support for caregivers of people with dementia [13], information on end-of-life issues that is available to caregivers of people with advanced dementia is scarce [10–12, 21]. Therefore, a German expert panel, with the help of laypeople, developed an educational booklet with several goals: increase family caregivers’ knowledge of the various palliative care issues, and encourage and aid family caregivers to become involved in medical and care decisions. The booklet contains relevant topics, such as the advanced stages of dementia, palliative care goals, the legal basis of decision making (the roles of the patient, the living will, the legal representative as well as the doctor), ethical considerations about life-prolonging measures, pharmacological and non-pharmacological treatment options for symptom relief, and dying and death. Finally**,** a checklist allows the reader to easily apply the topics of the booklet to his specific situation.

It seems that pre-existing informational guides mostly address caregivers of people with dementia who live in long-term care. Since many people with advanced dementia are cared for at home it is important to address these home-based caregivers as well [10]. Our booklet addresses these caregivers as well.

During the development of the booklet it became clear that it is impossible to merely translate current informative guides on the topic from other countries, e.g. Canada [14]. As van der Steen et al. have already stated, it is important to consider local ethical and legal aspects as well as the country-specific standards and practices when adapting advice and guidance on palliative care issues to a different country [22]. For the German booklet, the most important adaptions regarded legal issues, and the religious/ spiritual aspect.

In our trial we were able to show that family caregivers of patients with advanced dementia experienced a benefit from the booklet which they deemed comprehensible, concise and well-structured. Furthermore, it appeared to increase family caregivers’ knowledge and their level of involvement in decision making.

Our results match with previous findings of van der Steen et al. [23] who found that doctors and nurses in nursing homes anticipated that an informational booklet about comfort care in advanced dementia would be useful for families.

A surprisingly high number of caregivers had thought about individual palliative care issues before we provided them with the booklet. This fact was unexpected when the study was designed and created a measuring error. Even before provision of the booklet, more than half of the caregivers had already discussed palliative care issues with the medical and care team. Three quarters of the caregivers had made decisions about symptom relief and life-prolonging measures, almost two-thirds about the dying process but just over one-third about palliative care services. The vast majority of caregivers (84%) had already offered opinions, made suggestions and/ or expressed doubts regarding palliative care.

Caregivers were least informed about the topic “*Goals of palliative care; palliative/ hospice care services*”, which generally matched caregivers’ self-assessed knowledge of palliative goals and support from palliative care and hospice services. The topic had only been considered by less than half of the caregivers. We suppose this is due to the fact that palliative care for people with dementia in Germany has only recently come into clinical and research focus. The goals of palliative care and support from palliative care and hospice services have long been established for oncological diseases, yet are still somewhat neglected for dementia. Furthermore, the term “palliative” appears to still be reserved for the last days or weeks of life - not only by lay people but also by professional staff. Many caregivers in our study appeared surprised that palliative care issues apply to all people with advanced dementia, regardless of whether death is expected soon.

Although many caregivers had already thought about palliative care issues before our study, a considerable number of caregivers had not discussed these subjects or made decisions before participating in the study. Of these caregivers, up to 70% had made a decision after reading the booklet. Thus, the booklet supported and prompted the family caregivers of patients with advanced dementia to take an active role in decision making. However, only few of these caregivers had taken the next step to discuss palliative care issues with the medical and care team. The reasons for this are unclear, though we speculate that in many cases the patients’ disease course may not have been variable enough to warrant discussion within the short period between *Visit 1* and *Visit 2* (maximum 12 weeks).

Our study has some limitations.

1. The open study design, which lacks a control group, does not allow us to exclude other potential reasons for a gain in knowledge or decision making ability after provision of the booklet. Changes might be caused by other factors such as newspaper articles, discussions with friends and the disease progression that demands more consideration and decisions. However, the period between provision of the booklet and the second visit was as short as possible in order to exclude other sources of information as much as possible.
2. We did not investigate the effect of the booklet on caregiver burden and satisfaction with care. Many patient and caregiver related issues influence caregiver burden, and it is almost impossible to determine the impact of these factors on caregiver burden. The same is true for satisfaction with care. Furthermore, an informative guide might not have a positive effect on perception of care, rather just the opposite; it is possible that after reading the booklet, caregivers believe that their loved ones are not being treated optimally or that their opinions or suggestions are not taken seriously by the medical and care team.
3. We suspect an inclusion bias. Patient-caregiver dyads were recruited for this study from a university-based center of cognitive disorders, a tertiary care center, in a metropolitan area. Medical and care counseling of these dyads following diagnosis might have included palliative care issues, which might in part explain the high percentage of caregivers who had already been informed and worked through individual palliative care issues. Thus, the effect of any booklet should be tested on various caregivers, including less educated individuals and those from rural areas with potentially less counseling and support services available to them.
4. We did not conduct quizzes in order to objectify knowledge and knowledge gain of the caregivers, but assessed how the caregivers self-rated their knowledge. When designing the study, we considered results of a prior focus group with family caregivers who unanimously stated that they do not like to be questioned in studies as if it were an exam. Furthermore, it was impossible to determine whether the caregivers had really read the booklet. –
5. A participant bias in the sense of social desirability cannot be excluded. Overall, caregivers said they appreciated the project and the booklet very much. Perhaps some of them answered in a way to “support” the booklet as not to disappoint the investigator. We tried to minimize this factor by choosing an investigator who was not included in the booklet’s development.

## Conclusions

From our data we conclude that a simple and concise informative guide, a booklet on palliative care issues, is deemed very helpful by caregivers of patients with advanced dementia. Our results suggest that our booklet increases knowledge and prompts active decision making. For making this kind of support available to all those who need it, further testing and evaluation on a larger scare is necessary. The booklet was advertised to the palliative care services in Bavaria as well as to the regional and national Alzheimer Societies. The booklet is available for download on the webpage of the Bavarian Ministry for Health and Care https://www.bestellen.bayern.de/application/eshop_app000009?SID=1422726442&ACTIONxSESSxSHOWPIC(BILDxKEY:%27stmgp_pflege_047%27,BILDxCLASS:%27Artikel%27,BILDxTYPE:%27PDF%27. In only 8 months, 4000 booklets were downloaded or ordered as paper version. This underscores the strong need caregivers feel for informative material in the field of palliative care in advanced dementia.

## Supplementary information

**Additional file 1 Table 4.** Data collection. Interviews with the caregiver.

**Additional file 2 Table 5.** Caregivers’ self-rated knowledge about various palliative care knowledge prior and after provision of the booklet (1 = good; 2 = moderate; 3 = poor).

**Additional file 3 Table 6.** Participatory decision making of the family caregivers, modified version of the *Patients’ Perceived Involvement in Care Scale, Lerman* et al.*, 1990).*

**Additional file 4.** Questionnaire on the booklet‘s comprehensibility [19].

## Data Availability

The data that support the findings of this study are available on request from the corresponding author, [LR]. The data are not publicly available due to German data protection laws.

## Abbreviations

CDR: Clinical Dementia Rating Scale
MMSE: Mini Mental State Examination
PIC: Perceived Involement in Care Scale
WHO: World Health Organization
