# Supplementary material for: Development and testing of an informative guide about palliative care for family caregivers of people with advanced dementia
Source: BMC Palliat Care. 2020 Mar 12;19:30. doi: 10.1186/s12904-020-0533-3 (PMC7068859; doi:10.1186/s12904-020-0533-3)
Supplement: Supplementary file 1 — Additional file 1 Table 4. Data collection. Interviews with the caregiver. [file 12904_2020_533_MOESM1_ESM.docx]

Table 4. Data collection. Interviews with the caregiver

|  | Visit 1 | Visit 2 |
| --- | --- | --- |
| **Caregiver** |  |  |
| Demographics | x |  |
| Satisfaction and involvement of caregivers in care and treatment | | |
| Patients’ Perceived Involvement in Care Scale (PIC-Skala), modified for caregivers [16] | x | x |
| Participatory decision making and shared decision making in relation to palliative and hospice care at the end of life in dementia (How well do you regard your knowledge?) | x | x |
