# Supplementary material for: Development and testing of an informative guide about palliative care for family caregivers of people with advanced dementia
Source: BMC Palliat Care. 2020 Mar 12;19:30. doi: 10.1186/s12904-020-0533-3 (PMC7068859; doi:10.1186/s12904-020-0533-3)
Supplement: Supplementary file 2 — Additional file 2 Table 5. Caregivers’ self-rated knowledge about various palliative care knowledge prior and after provision of the booklet (1 = good; 2 = moderate; 3 = poor). [file 12904_2020_533_MOESM2_ESM.docx]

Table 5: Caregivers' self-rated knowledge about various palliative care knowledge prior and after provision of the booklet (1=good; 2=moderate; 3=poor)

|  | **Self-rated knowledge 1=good, 2=moderate, 3=poor** |
| --- | --- |
| 1. Symptoms, course of dementia, prognosis |  |
| 2. Goals of palliative care; palliative/ hospice care services |  |
| 3. Tasks of legal representative |  |
| 4. Life prolonging measures: tube feeding admission to hospital, resuscitation; consequences for the patient |  |
| 5. Pharmacological, non-pharmacological symptom relief (e.g. dyspnea, pain, agitation, anxiety, delirium) |  |
| 6. Dying, death (what is to be expected when the patient is dying) |  |
| **Sum Score** |  |
