# Supplementary material for: Development and testing of an informative guide about palliative care for family caregivers of people with advanced dementia
Source: BMC Palliat Care. 2020 Mar 12;19:30. doi: 10.1186/s12904-020-0533-3 (PMC7068859; doi:10.1186/s12904-020-0533-3)
Supplement: Supplementary file 3 — Additional file 3 Table 6. Participatory decision making of the family caregivers, modified version of the Patients’ Perceived Involvement in Care Scale, Lerman et al., 1990). [file 12904_2020_533_MOESM3_ESM.docx]

Table 6: Participatory decision making of the family caregivers, modified version of the *Patients' Perceived Involvement in Care Scale, Lerman et al., 1990)*

|  |  | **Yes=1, No=0** |
| --- | --- | --- |
|  | **1. I carefully thought about the following topics:** |  |
| **Considerations** | 1a. Symptoms, course of dementia, prognosis |  |
|  | 1b. Goals of palliative care; palliative/ hospice care services |  |
|  | 1c. Life prolonging measures: tube feeding admission to hospital, resuscitation; consequences for the patient |  |
|  | 1d. Pharmacological, non-pharmacological symptom relief (e.g. dyspnea, pain, agitation, anxiety, delirium) |  |
|  | 1e. Dying, death (what is to be expected when the patient is dying) |  |
| **Level of information exchange** | **2. I discussed following topics with the doctor/ care team:** |  |
|  | 2a. Symptoms, course of dementia, prognosis |  |
|  | 2b. Goals of palliative care; palliative/ hospice care services |  |
|  | 1c. Life prolonging measures: tube feeding admission to hospital, resuscitation |  |
|  | 2d. Pharmacological, non-pharmacological symptom relief (e.g. dyspnea, pain, agitation, anxiety, delirium) |  |
|  | 2e. Dying, death (what is to be expected when the patient is dying) |  |
| **Caregiver participation in decision making** | **3. I made a decision regarding the following topics:** |  |
|  | 3a. Goals of palliative care; palliative/ hospice care services |  |
|  | 3b. Life prolonging measures: tube feeding admission to hospital, resuscitation; consequences for the patient |  |
|  | 3c. Pharmacological, non-pharmacological symptom relief (e.g. dyspnea, pain, agitation, anxiety, delirium) |  |
|  | 3 d. Dying, death (what is to be expected when the patient is dying) |  |
|  | 4. I suggested the doctor/ care team a certain kind of treatment/ care |  |
|  | 5. I expressed doubts about treatment/care that the doctor/ care team suggested |  |
|  | 6. I gave my opinion (agreement or disagreement) about treatment and care |  |
