# Supplementary material for: Development and testing of an informative guide about palliative care for family caregivers of people with advanced dementia
Source: BMC Palliat Care. 2020 Mar 12;19:30. doi: 10.1186/s12904-020-0533-3 (PMC7068859; doi:10.1186/s12904-020-0533-3)
Supplement: Supplementary file 4 — Additional file 4. Questionnaire on the booklet‘s comprehensibility [19]. [file 12904_2020_533_MOESM4_ESM.docx]

Questionnaire on the booklet‘s comprehensibility [19]

We ask you to first evaluate the comprehensibility of each section of the booklet in Part 1. You can then evaluate the conciseness, simplicity and structure in Part 2.

Part 1: Please make a cross in each line according to your agreement to the statement

|  | **I agree very much** | **I agree** | **neither/**  **nor** | **I do not agree** | **I do not agree at all** |
| --- | --- | --- | --- | --- | --- |
| The **title** of the booklet is understandable. |  |  |  |  |  |
| The **aim** of the guidebook is presented in an understandable way (chapter 1). |  |  |  |  |  |
| The **symptoms of advanced dementia, disease progression and death** are presented in an understandable way (chapter 2). |  |  |  |  |  |
| The **goals and possibilities of palliative and hospice care** are presented in an understandable way (chapter 3). |  |  |  |  |  |
| What has to be decided with regard **to life-prolonging** measures, who does this and what the decisions are based on are clearly presented (page 7-9). |  |  |  |  |  |
| The **difficulties in making decisions** and possible options to seek support are presented in an understandable way (page 9-10). |  |  |  |  |  |
| **Artificial nutrition** and its disadvantages are presented in an understandable way (page 11-13). |  |  |  |  |  |
| The pros and cons of **hospital treatment** for intensive therapy are presented in an understandable way (page 13-14). |  |  |  |  |  |
| The procedure for **resuscitation** and possible consequences are presented in an understandable way (page 15). |  |  |  |  |  |
| The possibilities of **alleviating discomfort at the end of life** are presented in an understandable way (page 17-19). |  |  |  |  |  |
| The dying phase is described comprehensibly (chapter 6). |  |  |  |  |  |
| It is understandably described what happens **after death** (chapter 7). |  |  |  |  |  |
| The appendix, which contains frequently prescribed **medications**, is understandable. |  |  |  |  |  |
| The **checklist** in the appendix is helpful. |  |  |  |  |  |

Part 2: Please make a cross in each line at the place which seems most suitable to you

| **conciseness** | **+2** | **+1** | **0** | **-1** | **-2** | **verbosity** |
| --- | --- | --- | --- | --- | --- | --- |
| too short |  |  |  |  |  | too long |
| limited to the essentials |  |  |  |  |  | much insignificance |
| narrow |  |  |  |  |  | broad |
| focused |  |  |  |  |  | digressive |
| scarce |  |  |  |  |  | extensive |
| every word is necessary |  |  |  |  |  | many things could have been left out |

| **simplicity** | **+2** | **+1** | **0** | **-1** | **-2** | **complexity** |
| --- | --- | --- | --- | --- | --- | --- |
| simple representation |  |  |  |  |  | complicated presentation |
| short, simple sentences |  |  |  |  |  | long, complicated sentences |
| common words |  |  |  |  |  | unfamiliar words |
| technical terms explained |  |  |  |  |  | technical terms not explained |
| specific |  |  |  |  |  | conceptual |
| descriptive |  |  |  |  |  | undescriptive |

| **structure - order** | **+2** | **+1** | **0** | **-1** | **-2** | **missing structure - incoherence** |
| --- | --- | --- | --- | --- | --- | --- |
| coherent |  |  |  |  |  | incoherent |
| clear |  |  |  |  |  | confusing |
| good distinction between necessary and unnecessary information |  |  |  |  |  | poor distinction between necessary and unnecessary information |
| the train of thoughts remains visible |  |  |  |  |  | one often loses the train of thoughts |
| everything comes nicely in order |  |  |  |  |  | everything is mixed up |
